# Krabbe Disease: Report of a Rare Lipid Storage and Neurodegenerative Disorder

**DOI:** 10.7759/cureus.949

**Published:** 2017-01-01

**Authors:** Pratyusha Pavuluri, Sabitha Vadakedath, Rajkumar Gundu, Sushmitha Uppulety, Venkataramana Kandi

**Affiliations:** 1 Biochemistry, Chalmeda Anandrao Institute of Medical Sciences; 2 Biochemistry, Chalmeda Anand Rao Institute of Medical Sciences; 3 Department of Microbiology, Prathima Institute of Medical Sciences

**Keywords:** krabbe disease, autosomal recessive sphingolipidosis, progressive neurologic degeneration, galactosyl cerebroside, globoid cell leukodystrophy

## Abstract

Krabbe disease is a rare (one in 100,000 births) autosomal recessive condition, usually noticed among children. It causes sphingolipidosis (dysfunctional metabolism of sphingolipids) and leads to fatal degenerative changes affecting the myelin sheath of the nervous system. We report a case of a six-year-old male child who presented with symptoms of muscle spasticity and irritability. Diagnosis of this disease can only be made with clinical suspicion. Laboratory diagnosis includes brain magnetic resonance imaging (MRI), magnetic resonance (MR) spectroscopy, biochemical analysis of cerebrospinal fluid, and genetic analysis for detecting mutation in genes coding for galactosyl cerebroside (GALC). We report a case of late infantile Krabbe disease.

## Introduction

Krabbe disease or globoid cell leukodystrophy is a rare (one in 100,000 births), autosomal recessive, neurodegenerative disorder affecting the myelin sheath of the nervous system. It is a lipid storage disorder responsible for sphingolipidosis (dysfunctional metabolism of sphingolipids) usually noticed among children. Krabbe disease is characterized by defective functioning of lysosomal enzyme β-galactocerebrosidase (β-GALC) [1-2]. This enzyme is located on chromosome 14 and is responsible for accumulation of galactosyl ceramide. This produces a toxic compound psychosine, which damages white matter of the peripheral and central nervous system [3-4]. Based on the age of onset there are four forms of Krabbe disease, infantile, late infantile (very often parents ignore early signs), juvenile, and adult form [5-6]. Juvenile and adult forms are categorized as late onset Krabbe disease. The clinical classification of Krabbe disease is shown in Table 1.

**Table 1:** Clinical forms of Krabbe disease MRI - Magnetic resonance imaging. CSF - Cerebrospinal fluid.

Clinical forms of Krabbe disease	Radiological features and other biochemical parameters	Presenting symptoms	Morbidity/mortality
Infantile form (Diagnosed at first six months of life)	Brain MRI shows central demyelination with evidence of white matter disease and optic atrophy. CSF analysis shows raised protein levels.	Feeding difficulties, irritability, spasticity, hypertonia, hyperesthesia, blindness, deafness, peripheral demyelination causes progressive decline in psychomotor activity, limb weakness, ataxia, and seizures.	Death within few years of onset of the disease.
Late infantile form (Diagnosed between six months to three years of life)	Brain MRI shows high-intensity areas of demyelination in the brainstem and cerebellum. CSF analysis shows raised protein levels.	Normal early development is followed by paresthesias, decreased muscle strength, spasticity, ataxia, paresis, psychomotor arrest, psychomotor deterioration, optic atrophy, visual loss, macular cherry red spots, diminished head circumference, macrocephaly, and seizures.	Death within few years of onset of the disease.
Juvenile form (Diagnosed between 2 to 10 years of age)	Brain MRI normal in earlier stages and CSF analysis is usually normal.	Early normal development is followed by rapid psychomotor dysfunction, slower and progressive nerve degeneration, irritability, spasticity, ataxia. Seizures may be present.	Longer life with mild symptoms.
Adult form (Diagnosed after 10 years of age)	Brain MRI shows normal features in early stages and CSF analysis is usually normal.	Peripheral neuropathy, cerebellar dysfunction, impaired higher cortical functioning, irritability, spasticity, ataxia, seizures are present.	Significantly long life.

We present a case of late infantile type of Krabbe disease. The CAIMS Institutional Ethical Board approved this study and the approval number is IEC/CAIMS/2016-1113. Informed consent was obtained from the patient's family for this study.

## Case presentation

A six-year-old boy presented with symptoms that included shock-like jerky movements of the limbs, inability to hold his neck, functional urinary incontinence, and dependence on the mother for most daily activities. There was no visual deficit, with normal ophthalmoscopic examination. A physical examination revealed hypertonicity and hyperreflexes in both upper and lower limbs with a positive Babinski sign. He was unable to speak but otherwise appeared to have normal intellectual ability. The family history reveals a third degree consanguineous parents with a history of Krabbe disease. The other child of the same parent was observed to be normal. Initially, the patient's mother found a defective suckling of milk at birth, which was compensated by bottle feed and weaning. The milestones were normal until the age of two when the patient developed fever associated with seizures. This episode of seizure was misdiagnosed as a common febrile seizure. After this episode there was progressive spastic tetraparesis that made him unable to walk, talk, and caused difficulty in swallowing. The patient was reported to suffer from approximately twenty episodes of seizures a day. He had great difficulty in swallowing and had to be fed only a semi-solid and liquid diet.

A brain magnetic resonance imaging (MRI) showed demyelination in the brain stem and cerebellum. A brain magnetic resonance (MR) spectroscopy revealed elevated myo-inositol and choline-containing compounds with decreased N-aspartyl aspartate in the affected white matter area, and the grey matter showed neuronal degeneration. Mutational analysis by deoxyribonucleic acid (DNA) sequencing revealed a missense mutation on chromosome band 14q31.3, coding for GALC gene confirming Krabbe disease. The patient was advised to undergo physiotherapy to help maintain muscle tone and circulation. Cerebrospinal fluid evaluation, electroencephalography (EEG), and electromyography (EMG) tests could not be performed due to patient's concerns.

The patient was prescibed anticonvulsant drugs to minimize the seizure episodes and drugs to ease muscle spasticity.

## Discussion

**Figure 1:**
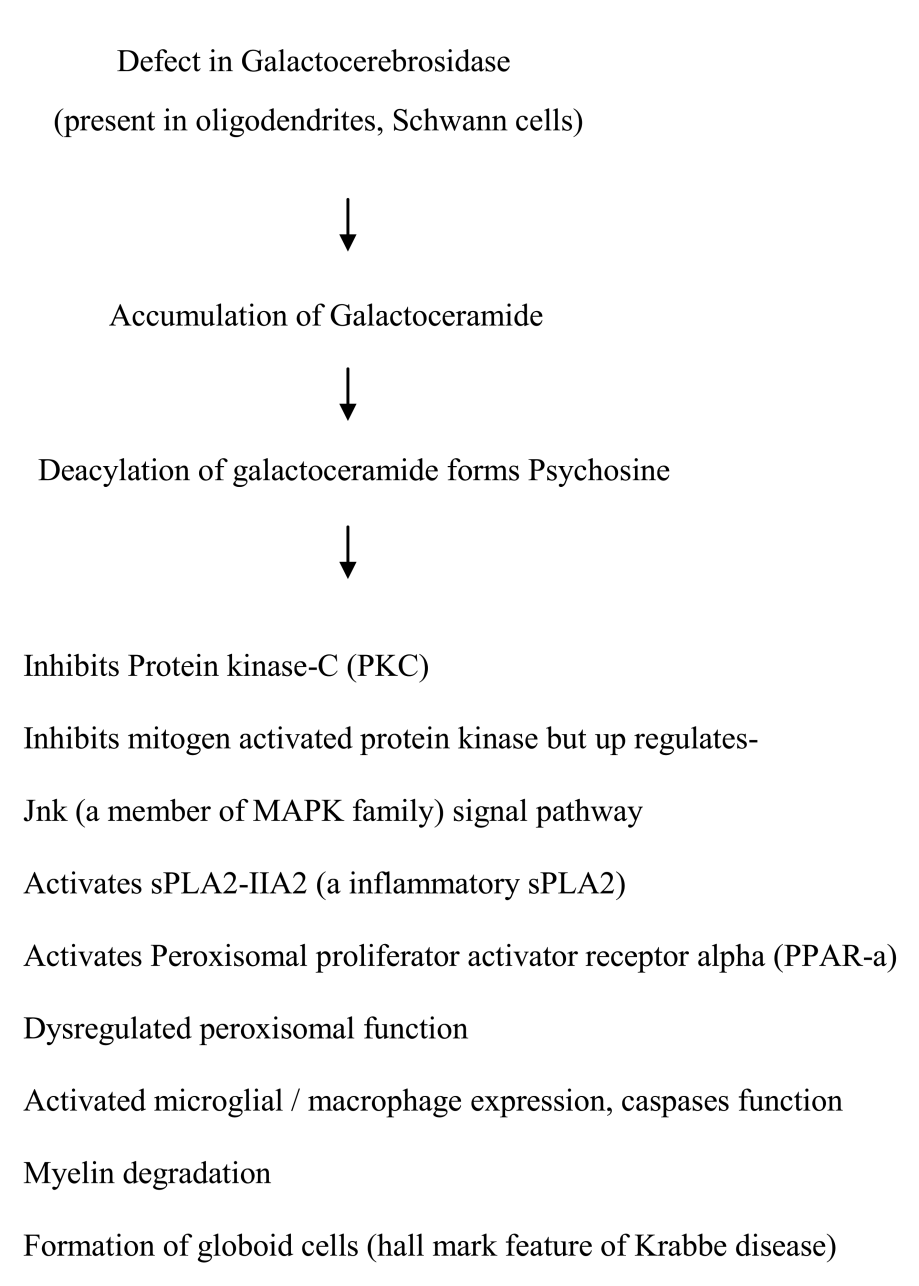
The pathway depicting consequences of mutation at chromosome 14

Krabbe disease occurs due to the deficiency of lysosomal enzyme galactocerebrosidase (expressed in oligodendrocytes and Schwann cells), which results in accumulation of a cytotoxic metabolite, psychosine (deacylated product of galactosylceramide). The accumulation of psychosine disrupts downstream biochemical pathways inhibiting activities of protein kinase C (PKC), mitogen-activated protein kinase (MAPK), dysregulates peroxisomal functions (inhibits plasmalogen synthesis required for myelin formation), activates caspases (apoptotic machinery), and thus, causes apoptosis of myelin-forming cells resulting in demyelination [7-8]. The pathway depicting dysfunctional GALC activity [2] is shown in Figure 1.

In the present case, a missense mutation was found on chromosome band 14q31.3, which was detected by gene sequencing analysis. Such mutations result in abnormal catalytic activity of lysosomes or defective hydrolase activity. It also causes misfolding of lysosomal proteins, leading to impaired transport of lysosomal enzymes into endoplasmic reticulum (ER), and a defective glycosylation in the golgi apparatus. The phagocytosis of myelin debris by macrophages results in the formation of globoid cells, which is the hallmark feature of Krabbe disease [9].

Krabbe disease in infants is fatal due to decline in psychomotor activity, and death occurs before two years. Late onset (juvenile and adult forms) disease has slow progression and the patient has a significantly longer life. The difference in severity of progression and age of onset of the disease is due to the difference in the mutational area of this gene and also turnover of the psychosine [9-10]. The majority of mutations in late onset disease occur at 5’-end whereas the severe infantile form is due to mutation occurring at 3’-end [10].

Krabbe disease needs to be differentially diagnosed with other neurodegenerative disorders including GM2 gangliosidoses (lysosomal lipid storage disorders caused by mutations in at least one of three recessive genes: HEXA, HEXB, and GM2A), Gaucher disease (a rare genetic disorder characterized by the deposition of glucocerebroside in cells of the macrophage-monocyte system, resulting in the deficiency of enzyme glucocerebrosidase), metachromatic leukodystrophy (progressive, inherited, and neurodegenerative disorders), and sphingomyelinase deficiency (Niemann-Pick disease (NPD) is a lipid storage disorder that results from the deficiency of enzyme, acid sphingomyelinase). X-linked adrenoleukodystrophy (X-ALD) (common among children between four and eight years, resulting in elevated plasma concentration of very-long-chain fatty acids (VLCFA)); Pelizaeus-Merzbacher disease (PMD) (affects the cortical white matter); and Alexander disease (affects the cortical white matter) should also be considered for differential diagnosis.

Although hematopoietic stem cell transplantation (HSCT) is seen as a potential treatment for Krabbe disease, previous research has observed that there is a varying degree of benefit with HSCT. Significant benefit of HSCT was noted in patients who were asymptomatic or mildly symptomatic and when transplanted within the first^ ^month of life, signifying the importance of diagnosis as early as possible after birth. Other treatment modalities being tried to treat Krabbe disease include enzyme replacement therapy, targeting inflammatory markers, gene therapy, and neural stem cell transplantation. Considering the fact that the patient was already showing neurological manifestations including quadriplegia, he was closely monitored and necessary medical care was provided.

## Conclusions

The present case highlights the importance of careful consideration of Krabbe disease among children with defective feeding habits. Although a feeding problem was noticed early in childhood in this case, it was ignored until the age of two years. Parents may be carriers of the gene responsible for Krabbe disease with no clinical symptoms, and clinical disease can be noted among children born to such parents. If suspected and diagnosed at the right time, stem cells from umbilical cord or bone marrow transplantation from an unrelated donor before onset of the symptoms may help in delaying neurological manifestations. The stem cells (embryonic) are unique because of their potential to develop into any type of cells and their ability to renew themselves. The use of stem cells in therapy is still in the phase of clinical trials, and much work has to be done before using it in the treatment. In conclusion, counselling families about the risk of disease and recommending prenatal testing in future pregnancies could help early diagnosis of Krabbe disease. To reduce the mortality and morbidity, necessary care by a neurologist, ophthalmologist, occupational therapist, audiologist, and physiotherapist is recommended.
